# Synthesis and Characterization of Fully Conjugated Ladder Naphthalene Bisimide Copolymers

**DOI:** 10.3390/polym10070790

**Published:** 2018-07-18

**Authors:** Feng Liu, Yonggang Wu, Chao Wang, Junshu Ma, Fan Wu, Ye Zhang, Xinwu Ba

**Affiliations:** College of Chemistry and Environmental Science, Hebei University, Baoding 071002, China; liufengzz27@163.com (F.L.); 15209828399@163.com (C.W.); fengzhiwangzhe@126.com (J.M.); 18830268213@163.com (F.W.); azhangye0817@163.com (Y.Z.)

**Keywords:** NDI, carbazole, thieno[3,4-*b*]thiophene, imine-bridged, Ladderized

## Abstract

Fully conjugated ladder copolymers have attracted considerable attention due to their unique fused-ring structure and optoelectronic properties. In this study, two fully conjugated ladder naphthalene diimide (NDI) copolymers, P(NDI-CZL) and P(NDI-TTL) with imine-bridged structures are presented in high yields. Both of the two copolymers have good solubility and high thermal stability. The corresponding compounds with the same structure as the copolymers were synthesized as model system. The yields for each step of the synthesis of the model compounds are higher than 95%. These results suggest that P(NDI-CZL) and P(NDI-TTL) can be synthesized successfully with fewer structural defects. The structures and optoelectronic properties of compounds and copolymers are investigated by NMR, fourier transform infrared spectroscopy (FTIR), ultraviolet-visible spectroscopy (UV-vis), and cyclic voltammetry (CV). Both in solution and as a thin film, the two copolymers show two UV-vis absorption bands (around 300–400 nm and 400–750 nm) and a very weak fluorescence. The collective results suggest that the two fully conjugated ladder copolymers can be used as potential acceptor materials.

## 1. Introduction

Recently, fully conjugated ladder polymers have been leaded to rapid development in the field of organic optoelectronic materials due to their unique fused structure and excellent properties [1,2,3,4,5,6,7,8,9]. The main chain on the fully conjugated ladder polymer is π-conjugated and fused structure [10,11,12,13,14]. This structure can improve π-electron delocalization and create the unique properties [15,16,17,18,19], such as high thermal and chemical stability, narrow band gaps, and high charge-carrier mobility. Although various synthesis methods have been used to synthesis fully conjugated ladder polymers [20,21,22,23,24,25,26,27,28,29], there are still some difficulties (such as structure defects or solution-processability) which need to be overcome to improve the properties of ladder polymers [30,31,32,33,34,35].

Introduction of electron-rich donor units and electron-deficient acceptor units into the main backbone chain of fully conjugated ladder polymer can offer an effective way to improve the ladder polymer’s properties [36,37,38,39,40]. Naphthalene diimide (NDI) units and perylene diimide (PDI) units demonstrate unique properties, such as high electron affinity, π-stacking behavior, and good absorption. Therefore, NDI units and PDI units are often used as acceptor units [41,42]. Compared to PDI-based copolymers, NDI-based copolymers have better regional regularity and a stronger intermolecular interaction which can improve the π-stacking behavior and electron mobility. Recent NDI-based copolymers contain different donor units such as thiophene (T), selenophene (Se), bithiophene (BT), and thieno[3,2-*b*]thiophene (TT), have been reported to have excellent properties [43,44,45,46,47,48,49,50]. But, few reports exist on fully conjugated ladder NDI-based polymers, which may be due to their limited solubility and structural defects. In 2011, Durban et al. first reported imine-bridged fully conjugated ladder NDI-based polymer structures [51]. The ladder polymer shows good solution-processability and has an electron mobility of 0.0026 cm^2^ V^−1^·s^−1^.

In this paper, we design a new synthesis method with the aim to obtain NDI-based fully conjugated ladder polymer free of structure defects. The two fully conjugated ladder polymers were constructed by acceptor unit (NDI) and donor unit (carbazole and thieno[3,4-*b*]thiophene). Meanwhile, the corresponding compounds with the same structures as the copolymers were synthesized as model systems. The two ladder polymers with imine-bridged structures were treated by CH_3_COOH and presented in high yields. The imine-bridged structure was confirmed by NMR, FTIR, UV-vis, and cyclic voltammetry (CV). This work will be beneficial to future research investigating the performance and optimization of organic photovoltaic devices.

## 2. Materials and Methods

### 2.1. Materials

Commercial chemicals were used without further purification. Tetrahydrofuran (THF) and dichloromethane (DCM) were distilled by a standard process before using. The reactions were monitored by thin layer chromatography (TLC) with silica gel 60 F254 (Merck, 0.2 mm, Darmstadt, Germany).

### 2.2. Instrumentation

^1^H and ^13^C NMR data were acquired on a Bruker AV600 spectrometer. The electrochemical behavior was recorded by cyclic voltammetry (Ivium Plus II, Ivium Technologies BV, Eindhoven, The Netherlands) with a standard three-electrode electrochemical cell, consisting of a glassy carbon working electrode, a Pt wire counter electrode, and an Ag/Ag^+^ (0.01 M in CH_3_CN) reference electrode. UV-visible absorption spectra were obtained on a Shimadzu UV-visible spectrometer (UV-2550, Shimadzu Corp., Tokyo, Japan). Fluorescence spectra were investigated by a Shimadzu fluorescence spectrophotometer (RF-5301PC, Shimadzu Corp., Tokyo, Japan). MALDI-TOF analyses were obtained by a Bruker Daltonics Inc. Autoflex III (Billerica, MA, USA). Thermal gravimetric analysis (TGA) and differential scanning calorimetry (DSC) measurements were carried out under nitrogen on a Perkin-Elmer Pyris 6 TGA (Perkin-Elmer, Waltham, MA, USA) (heating rate of 10 °C/min) and Perkin-Elmer Diamond DSC instruments (Perkin-Elmer, Waltham, MA, USA) (scanning rate of 10 °C/min), respectively.

### 2.3. General Synthesis for Compound NDI-CZL and Polymer P(NDI-CZL)

**Scheme 1:**
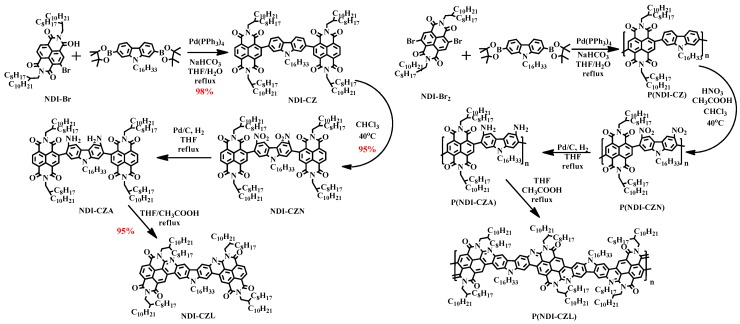
The synthetic routes to compound NDI-CZL and polymer P(NDI-CZL).

The fully ladder-type conjugated compound and copolymer were synthesized in four steps (as shown Scheme 1). First, the compound NDI-CZ and copolymer P(NDI-CZ) were synthesized by naphthalene diimide and 2,7-bis(4,4,5,5-tetramethyl-1,3,2-dioxaborolan-2-yl)-9-hexadecyl-9*H*-carbazole, using Suzuki coupling conditions and Pd(PPh_3_)_4_ as a catalyst. Afterward, HNO_3_ was used in addition to the compound and copolymer to introduce a –NO_2_ group. The –NO_2_ group was further converted into the corresponding amine derivative through Pd/C-catalyzed hydrogenation. Last, the carbonyl groups reacted with adjacent amine groups that can form intramolecular imine-bridged fused structures, using CH_3_COOH as catalyst. The model compound NDI-CZL was synthesized successfully in high yield which then permitted the synthesis of the fully ladder-type conjugated copolymers, P(NDI-CZL). The details of the synthesis for compounds and copolymers can be found in the supporting information. The compounds and copolymers were characterized in detail by NMR and MALDI-TOF, see the Supporting Information.

### 2.4. General Synthesis for Compound NDI-TTL and Polymer P(NDI-TTL)

**Scheme 2:**
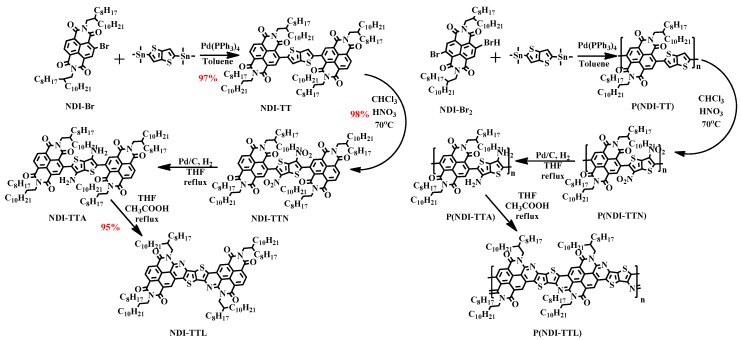
The synthetic routes to compound NDI-TTL and polymer P(NDI-TTL).

The ladder-type conjugated compound and copolymer were synthesized in four steps (as shown in Scheme 2). First, the compound NDI-TT and copolymer P(NDI-TT) were synthesized by naphthalene diimide and 2,5-bis(trimethylstannyl)thieno[3,2-*b*]thiophene, using Stille coupling conditions and Pd(PPh_3_)_4_ as catalyst. Then, HNO_3_ was used in addition to the compound and copolymer to introduce –NO_2_ group. The –NO_2_ group was further converted into the corresponding amine derivative through Pd/C-catalyzed hydrogenation. Last, the carbonyl groups reacted with adjacent amine groups that can form intramolecular imine-bridged fused structures, using CH_3_COOH as catalyst. The model compound NDI-TTL was synthesized successfully in high yield, allowing the synthesis the ladder-type conjugated copolymers, P(NDI-TTL). The details of the synthesis for compounds and copolymers are seen in the supporting information. The compounds and copolymers were characterized by NMR and MALDI-TOF, see Supporting Information.

## 3. Results

### 3.1. Synthetic Route Discussion

To ensure the synthetic route can successfully synthesize the fully conjugated ladder copolymers with fewer structure defects, first we use model compounds to discuss the synthetic routes. The compounds NDI-CZ, NDI-CAN, and NDI-CZL were synthesized in high yields; 98%, 95%, and 95%, respectively. The compounds NDI-TT, NDI-TTN, and NDI-TTL were synthesized in high yields; 97%, 98% and 95%, respectively. The NMR spectrum and MALDI-TOF were used to identify the structure of target compounds. The successful synthesis of the fully conjugated ladder copolymers was made feasible by; (1) the simplicity of the synthesis route; (2) the determined structure; and (3) the high yields obtained at every step.

### 3.2. SEC Trace for Copolymers

**Figure 1:**
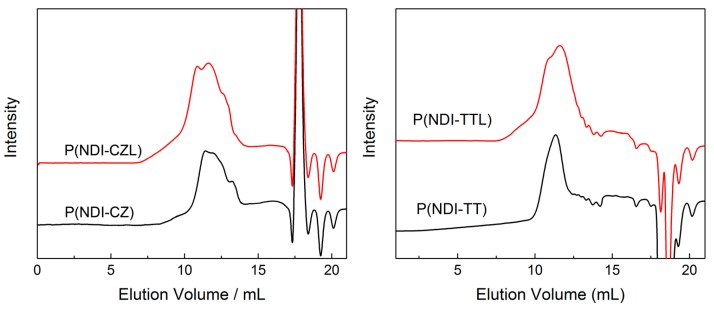
The SEC chromatograms of polymers.

The number average molecular weight (*M_n_*) of the copolymers was investigated by size-exclusion chromatography (SEC) in THF (polystyrene standards) (as shown in Figure 1). All data are listed in Table 1. The *M_n_* values of P(NDI-CZ), P(NDI-CZL), P(NDI-TT), and P(NDI-TTL) were 16.4 × 10^3^ g/mol, 18.9 × 10^3^ g/mol, 20.2 × 10^3^ g/mol, and 26.0 × 10^3^ g/mol, respectively, and the polydispersity index (PDI) values were 1.62, 1.78, 1.50 and 1.76, respectively. The increased molecular weights of P(NDI-CZL) and P(NDI-TTL) can be recognized as being overestimations due to the rigidity structure, rather than the actual molecular weight. The planarity the structure after ladderization reaction will effectively increase the hydrodynamic radius of copolymers in solution.

### 3.3. Thermal Properties of the Copolymers

**Figure 2:**
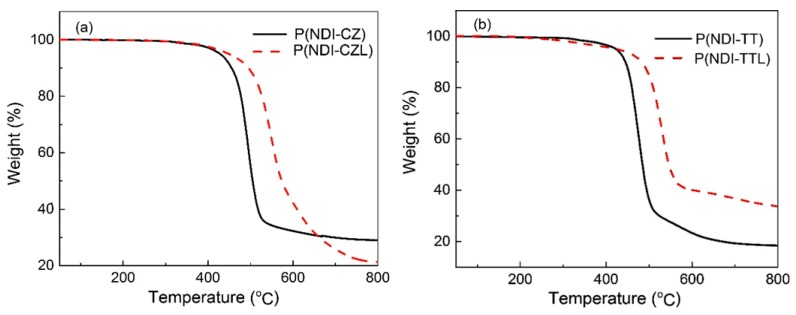
The thermogravimetric analysis (TGA) traces of the polymers. (**a**) for P(NDI-CZ), P(NDI-CZL) and (**b**) for P(NDI-TT), P(NDI-TTL).

**Figure 3:**
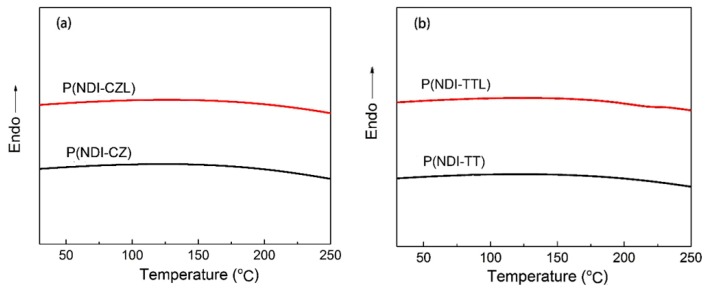
The differential scanning calorimetry (DSC) thermograms of the polymers. (**a**) for P(NDI-CZ), P(NDI-CZL) and (**b**) for P(NDI-TT), P(NDI-TTL).

The thermal properties of the copolymers were acquired by thermal gravimetric analysis (TGA) (as shown in Figure 2) and differential scanning calorimetry (DSC), (as shown in Figure 3). The decomposition temperatures (with a 5% mass loss) for copolymers P(NDI-CZ), P(NDI-CZL), P(NDI-TT), and P(NDI-TTL) were 430 °C, 450 °C, 415 °C, and 420 °C, respectively. All the decomposition temperatures of the copolymers were above 410 °C, indicate that the copolymers have an excellent thermal stability. After ladderization reaction, the decomposition temperatures of copolymers were increased. In the heating and cooling DSC scans, there were no apparent glass transition processes or other thermal processes. This phenomenon suggested that the copolymers have amorphous structures.

### 3.4. FTIR Spectra of Copolymers

**Figure 4:**
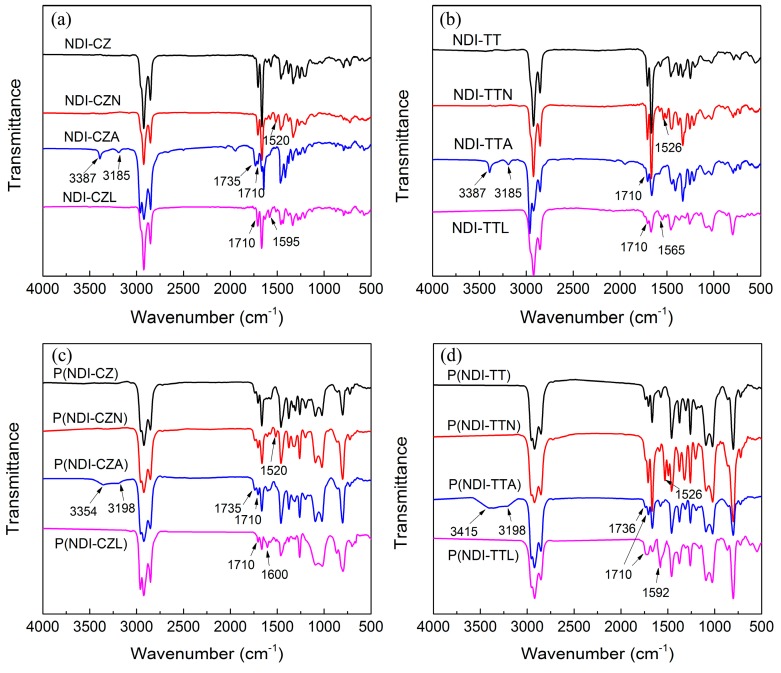
FTIR Spectra of compounds and copolymers before and after ladderization. (**a**,**b**) for compounds; (**c**,**d**) for copolymers.

Furthermore, the imine-bridged structure was confirmed by the FTIR spectrum (shown in Figure 4). After the nitration reaction, the –NO_2_ stretch peak around 1520 cm^−1^ appeared in both compounds NDI-CZN, NDI-TTN and copolymers P(NDI-CZN), P(NDI-TTN), compared to NDI-CZ, NDI-TT, P(NDI-CZ) and P(NDI-TT), respectively. Then the Pd/C-catalyzed hydrogenation reaction, the loss of –NO_2_ stretch peak and two –NH_2_ stretch peaks appeared (3387 and 3185 cm^−1^ for NDI-CZA, 3354 and 3198 cm^−1^ for P(NDI-CZA), 3387 and 3185 cm^−1^ for NDI-TTA, 3415 and 3198 cm^−1^ for P(NDI-TTA), respectively). The completely disappears of the –NO_2_ stretch peak means the Pd/C-catalyzed hydrogenation is thoroughly effective. Last, after the ladderization reaction which formed the imine-bridged structures, the two –NH_2_ stretch peaks and the –C=O stretch peak (around 1736 cm^−1^) completely disappeared and a –C=N stretch peak appeared around 1590 cm^−1^.

### 3.5. Photophysical Properties of Compounds and Copolymers

**Figure 5:**
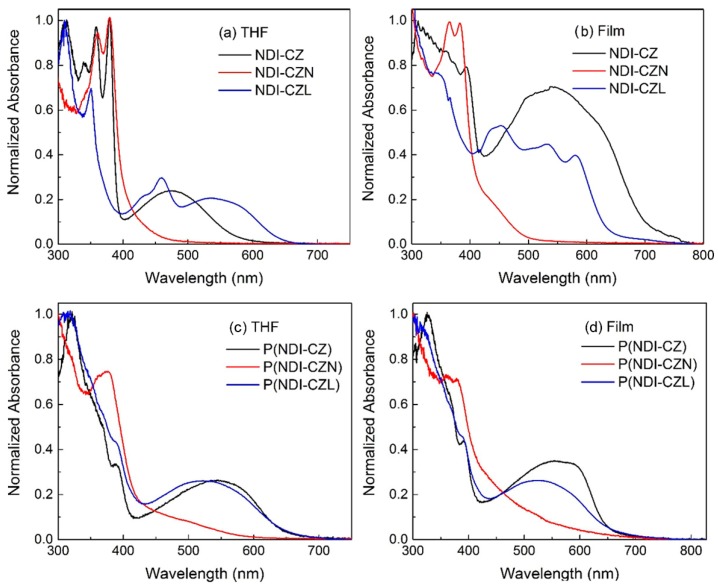
(**a**,**c**) Solution UV-vis absorption spectra; (**b**,**d**) thin-film UV-vis absorption spectra.

The UV-vis absorption spectra of the three compounds and copolymers in dilute THF solutions and thin films are shown in Figure 5. Compound NDI-CZ shows two distinct UV-vis absorption bands, around 300–400 nm and 400–700 nm. The absorption band around 300–400 nm was assigned to π-π* transitions in the molecular backbone. The absorption band around 400–700 nm was assigned to intramolecular charge transfer (ICT) between donor unit (CZ) to acceptor unit (NDI). After the nitration reaction, –NO_2_ group was introduced into the molecular backbone. As the –NO_2_ group is a strong electron-withdrawing group which can destroy the balance of the intramolecular charge transfer in the molecular backbone, the absorption band around 400–700 nm disappears. After the structural transformation in which the imine-bridge structure is forming, the intramolecular charge transfer between donor unit and acceptor unit reappears. Therefore, there are reappearance corresponding absorption band around 400–650 nm.

The copolymers show a similar UV-vis absorption to the compounds. Copolymer P(NDI-CZ) was a typical donor-acceptor (D-A) type polymer with two distinct UV-vis absorption bands, around 300–400 nm and 400–700 nm. The copolymer P(NDI-CZN) has only one UV-vis absorption band, around 300–450 nm. Copolymer P(NDI-CZL) shows two UV-vis absorption bands, around 300–420 nm and 420–700 nm.

Moreover, compared to the solution absorption, the thin-film absorption of the compound NDI-CZ and copolymers P(NDI-CZ) showed slight red-shifting due to the interchain aggregation and π-π stacking. While, due to the fused-ring constitution restricts the free torsional motion, the thin-film absorption of the final fully conjugated ladder compound NDI-CZL and the copolymer P(NDI-CZL) show no obvious red-shifting compared to the solution absorption.

**Figure 6:**
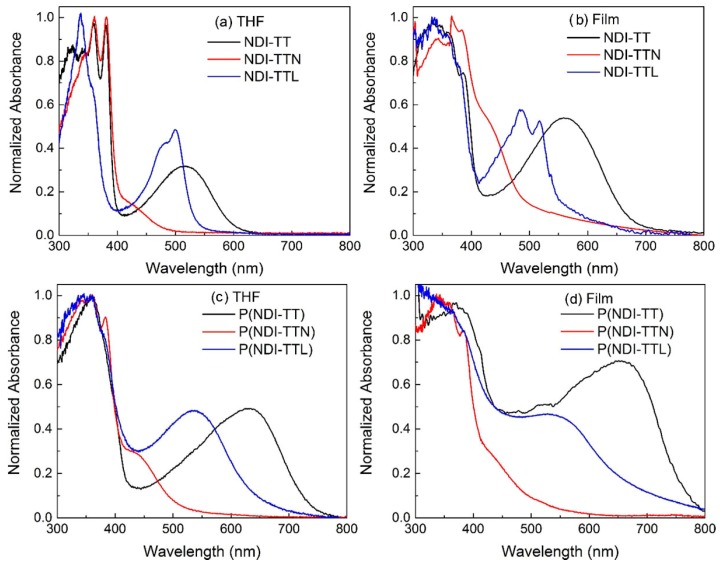
(**a**,**c**) Solution UV-vis absorption spectra; (**b**,**d**) thin-film UV-vis absorption spectra.

Figure 6 showed the UV-vis absorption spectra of the compounds and copolymers in dilute THF solutions and as thin films. Compound NDI-TT showed two distinct UV-vis absorption bands, around 300–400 nm and 400–600 nm. The absorption band around 300–400 nm is assigned to π-π* transitions in the molecule backbone. The absorption band around 400–600 nm is assigned to intramolecular charge transfer (ICT) between donor unit (TT) to acceptor unit (NDI). After the nitration reaction, –NO_2_ group was introduced into the molecular backbone. As –NO_2_ group is a strong electron-withdrawing group which can destroy the balance of the intramolecular charge transfer in the molecular backbone, the absorption band around 400–600 nm disappears. After the structural transformation in which the imine-bridged structure is formed, the intramolecular charge transfer between the donor unit and acceptor unit reappeared. Therefore, the absorption band around 400–560 nm reappeared.

The copolymers show a similar UV-vis absorption to the compounds. Copolymer P(NDI-TT) was a typical D-A type polymer with two distinct UV-vis absorption bands, around 300–420 nm and 420–750 nm. The copolymer P(NDI-TTN) has only one UV-vis absorption band, around 300–500 nm. Copolymer P(NDI-TTL) shows two UV-vis absorption bands, around 300–420 nm and 420–670 nm.

Moreover, compared to the solution absorption, the thin-film absorptions of the compound NDI-TT and copolymers P(NDI-TT) showed red-shifting due to the interchain aggregation and π-π stacking. While the fused-ring constitution restricts free torsional motion, the thin-film absorption of the final fully conjugated ladder compound NDI-TTL and copolymer P(NDI-TTL) showed no obvious red-shifting compared to the solution absorption.

### 3.6. Electrochemical Properties of Copolymers

**Figure 7:**
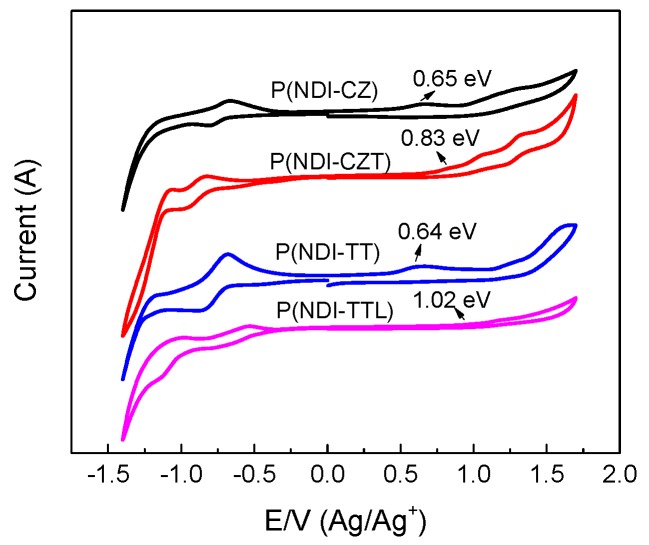
The cyclic voltammograms of polymers (0.1 M tetrabutylammonium hexafluorophosphate (TBAPF6) in CH_2_Cl_2_ solution, the scan rate was 30 mV·s^−1^).

The electrochemical properties of ladder-type conjugated copolymers and model compounds were performed by using cyclic voltammetry in a three-electrode cell at room temperature with Ag/Ag^+^ as the reference electrode and Fc/Fc^+^ redox system as the internal standard. It is assumed that the redox potential of Fc/Fc^+^ has an absolute energy level of −4.80 eV compared to a vacuum. The CH_2_Cl_2_ solution contains 0.1 M tetrabutylammonium hexafluorophosphate (TBAPF6). The scan rate was 30 mV·s^−1^ in the range of −1.4–1.7 V. Figure 7 shows the CV curves of the copolymers. The energy levels of the highest occupied molecular orbital (HOMO) were calculated by *E_HOMO_* = −(*Φ_ox_* + 4.8) eV. Band gap was calculated from the onset of the absorption spectra, Δ*E_gap_* = 1240/λ. The lowest unoccupied molecular orbital (LUMO) level of the polymers was calculated by *E_LUMO_* = *E_HOMO_* + Δ*E_gap_*. All the result data of the copolymers were listed in Table 1. The HOMO values of copolymers P(NDI-CZL) and P(NDI-TTL) were −5.47 eV and −5.68 eV, respectively. The LUMO values of copolymers P(NDI-CZL) and P(NDI-TTL) were −3.62 eV and −3.85 eV, respectively.

## 4. Conclusions

Two fully conjugated ladder NDI-based copolymers, P(NDI-CZL) and P(NDI-TTL) with imine-bridge structure, have been presented. The imine-bridge formation structure was confirmed by FTIR, MALDI-TOF, and UV-vis. The fully ladder-conjugated copolymers exhibit good solution-processability. The *M_n_* values were 18.9 × 10^3^ g/mol and 26.0 × 10^3^ g/mol for P(NDI-CZL) and P(NDI-TTL), respectively. The fully ladder-conjugated copolymers P(NDI-CZL) and P(NDI-TTL) show two distinct UV-vis absorption bands, which are assigned to π-π* transitions in the molecule backbone and ICT absorption band, respectively. The HOMO values were −5.47 eV and −5.68 eV for P(NDI-CZL) and P(NDI-TTL), respectively. The TGA results showed that all copolymers have the desirable thermostable properties. This initial study provides insight for the rational design of fully ladder-conjugated copolymers and the results show that the copolymers possess great potential as acceptor materials.

## Figures and Tables

**Table 1:** Polymer properties and yields.

	Yield %	*M_n_* × 10^3^ g/mol ^a^	*M_w_* × 10^3^ g/mol ^a^	PDI ^a^	T/°C ^b^	HOMO/eV ^c^	LUMO/eV ^d^	Δ*E_gap_*/eV ^e^
P(NDI-CZ)	96	16.4	26.5	1.62	430	−5.24 (±0.10)	−3.38	1.86
P(NDI-CZL)	93	18.9	33.7	1.78	455	−5.47 (±0.10)	−3.62	1.85
P(NDI-TT)	96	20.2	30.1	1.50	415	−5.25 (±0.10)	−3.62	1.63
P(NDI-TTL)	93	26.0	45.7	1.76	422	−5.68 (±0.10)	−3.85	1.83

^a^ Determined by GPC; ^b^ determined from thermogravimetric analysis (TGA), T was the temperature when 5% weight loss had occurred; ^c^ measured by cyclic voltammetry (CV), *E_HOMO_* = −(*Φ_ox_* + 4.8) eV (highest occupied molecular orbital (HOMO)); ^d^ calculated by *E_LUMO_* = *E_HOMO_* + Δ*E_gap_* (lowest unoccupied molecular orbital (LUMO)); ^e^ band gap calculated from the onset of the absorption in the UV-vis spectra, Δ*E_gap_* = 1240/λ.

## Supplementary Materials

The following are available online at http://www.mdpi.com/2073-4360/10/7/790/s1, Optical properties of products, ^1^H and ^13^C NMR and FTIR spectra. Figure S1: The cyclic voltammograms of compounds. Table S1: The CV date of compounds.
