# Supplementary material for: Synthesis and Characterization of Fully Conjugated Ladder Naphthalene Bisimide Copolymers
Source: Polymers (Basel). 2018 Jul 18;10(7):790. doi: 10.3390/polym10070790 (PMC6403639; doi:10.3390/polym10070790)
Supplement: Supplementary file 1 [file polymers-10-00790-s001.pdf]

Supplementary Files

Synthesis and Characterization of Fully Conjugated Ladder Naphthalene Bisimide Copolymers

Materials and Instruments

Commercial chemicals were used without further purification. Tetrahydrofuran (THF), and dichloromethane (DCM) were distilled by a standard process before using. The reactions were monitored by thin layer chromatography (TLC) with silica gel 60 F254 (Merck, 0.2 mm). ^{1}H and ^{13}C NMR data were acquired on a Bruker AV600 spectrometer. The electrochemical behavior was recorded by cyclic voltammetry (Holland, Ivium Plus II) with a standard three-electrode electrochemical cell. A glassy carbon working electrode, a Pt wire counter electrode, and an Ag/Ag^{+} (0.01 M in CH_3CN) reference electrode. UV-visible absorption spectra were obtained on a Shimadzu UV-visible spectrometer model UV-2550. Fluorescence spectra were investigated by a Shimadzu RF-5301PC fluorescence spectrophotometer. MALDI-TOF analyses were obtained by Bruker Daltonics Inc Autoflex III. Thermal gravimetric analysis (TGA) and differential scanning calorimetry (DSC) measurements were carried out under nitrogen on Perkin-Elmer Pyris 6 TGA (heating rate of 10 °C/min) and Perkin-Elmer Diamond DSC instruments (scanning rate of 10 °C/min), respectively, to record TGA and DSC curves.

Experimental Section

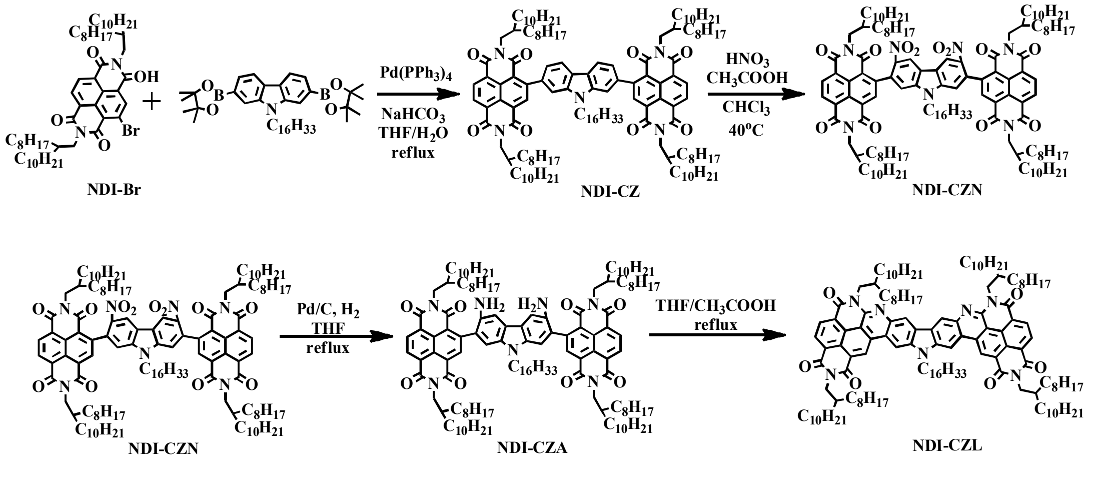

Scheme S1. The synthetic routes to compound NDI-CZL.

Compound NDI-CZ

A mixture of NDI-Br (1.81 g, 2.00 mmol), 2,7-bis(4,4,5,5-tetramethyl-1,3,2-dioxaborolan-2-yl)-9 -hexadecyl-9H-carbazole (0.60 g, 0.93 mmol), $NaHCO_3$ (0.35 g), THF (25 mL) and H_2O (4 mL) was degassed, $Pd(PPh_3)_4$ (20 mg, 0.018 mmol) was added under a nitrogen atmosphere. The mixture was heated by microwave reactor at reflux and stirred under nitrogen for 3 h. After cooling, water and CH_2Cl_2 (200 mL) were added, the organic layer was separated and dried over Na_2SO_4. After removal of the solvent, the residue was chromatographically purified on silica gel eluting with petroleum ether/dichloromethane (v/v = 1/2) to afford compound NDI-CZ as a dark violet solid (1.86 g, 98%). ^{1}H NMR (600 MHz, $CDCl_3$, ppm): δ = 8.85 (d, J = 7.54 Hz, 2H), 8.79 (d, J = 6.56 Hz, 4H), 8.22 (d, J = 7.86 Hz, 2H), 7.47 (s, 2H), 7.25 (s, 2H), 4.32 (s, 2H), 4.17 (d, J = 7.00 Hz, 4H), 4.05 (d, J = 6.80 Hz, 4H), 2.00 (m, 2H), 1.93 (m, 4H), 1.23 (m, 154H), 0.85 (t, J = 7.04 Hz, 27H); ^{13}C NMR (150 MHz, $CDCl_3$, ppm): δ 163.01, 160.68, 143.10, 138.23, 133.83, 130.45, 127.86, 125.84, 122.99, 111.89, 108.27, 104.37, 94.57, 90.47, 84.51, 82.96, 72.77, 64.65, 42.71, 42.46, 36.48, 30.12, 29.65, 29.34, 26.50, 22.68, 22.66, 14.09.

Compound NDI-CZN

A mixture of NDI-CZ (1.80 g, 0.881 mmol), concentrated nitric acid/acetic acid (2 mL, *v*/*v* = 1/3) and chloroform (10 mL) was heated at 40 °C for 8 h. After cooling, water and CH_2Cl_2 (200 mL) were added, the organic layer was separated and dried over Na_2SO_4. After removal of the solvent, the residue was chromatographically purified on silica gel eluting with petroleum ether/dichloromethane (*v*/*v* = 1/2) to afford compound NDI-CZN as a yellow solid (1.79 g, 95%). ^{1}H NMR (600 MHz, $CDCl_3$, ppm): δ = 9.28 (s, 2H), 8.85 (d, *J* = 7.62 Hz, 2H), 8.83 (d, *J* = 7.62 Hz, 2H), 8.62 (d, *J* = 10.82 Hz,2H), 7.30 (s, 2H), 4.31 (s, 2H), 4.17 (d, *J* = 7.00 Hz, 4H), 3.98 (d, *J* = 6.80 Hz, 4H), 2.01 (m, 2H), 1.86 (m, 4H), 1.24 (m, 157H), 0.85 (t, *J* = 7.04 Hz, 27H); ^{13}C NMR (150 MHz, $CDCl_3$, ppm): δ 163.14, 163.06, 162.87, 162.83, 162.67, 145.24, 144.16, 141.16, 141.33, 136.68, 133.68, 133.21, 133.14, 131.75, 131.63, 131.01, 126.87, 126.64, 126.46, 125.95, 122.32, 119.50, 110.14, 109.96, 72.43, 59.79, 54.75, 45.13, 44.78, 36.77, 36.45, 31.89, 31.62, 31.54, 31.40, 29.96, 29.42, 29.19, 27.29, 26.44, 26.36, 26.25, 22.68, 14.09.

Compound NDI-CZA

A mixture of NDI-CZN (1.50 g, 0.71 mmol), Pd/C (70 mg) and THF (10 mL) was heated at reflux and stirred under H_2 for overnight. After cooling, Pd/C was removed by filtration, the solution was collected and removed to get the crude black product NDI-CZA (1.43 g, 97%). The compound NDI-CZA was directe to the next step without further purified.

Compound NDI-CZL

A mixture of NDI-CZA, acetic acid (2 mL) and THF (10 mL) was heated at reflux and stirred under nitrogen for overnight. After cooling, water and CH_2Cl_2 (200 mL) were added, the organic layer was separated and dried over Na_2SO_4. After removal of the solvent, the residue was chromatographically purified on silica gel eluting with petroleum ether/dichloromethane (*v*/*v* = 1/4) to afford compound NDI-CZL as a brownish red solid (1.37 g, the total yield of two steps was 95%). ^{1}H NMR (600 MHz, $CDCl_3$, ppm): δ = 9.28 (s, 2H), 8.51 (m, 6H), 7.93 (s, 2H), 4.89 (m, 4H), 4.65 (m, 2H), 4.31 (m, 4H), 2.26 (m, 2H), 2.20 (m, 2H), 2.07 (m, 4H), 1.24 (m, 157H), 0.81 (t, *J* = 7.04 Hz, 27H); ^{13}C NMR (150 MHz, $CDCl_3$, ppm): δ 163.51, 163.20, 160.92, 145.12, 141.07, 139.34, 130.65, 129.54, 128.38, 128.34, 127.08, 126.55, 125.99, 124.27, 124.24, 124.08, 124.05, 123.74, 123.46, 121.00, 110.14, 105.26, 105.01, 104.49, 98.39, 45,79, 45.32, 37.08, 36.58, 31.93, 31.85, 30.18, 29.74, 29.61, 29.38, 29.31, 26.73, 22.68, 26.64, 22.60, 14.08, 14.05, 14.00; MS (MALDI-TOF): m/z: calcd for $C_{136}H_{207}N_7O_6$: 2036.2 $[M]^+$; found: 2036.1.

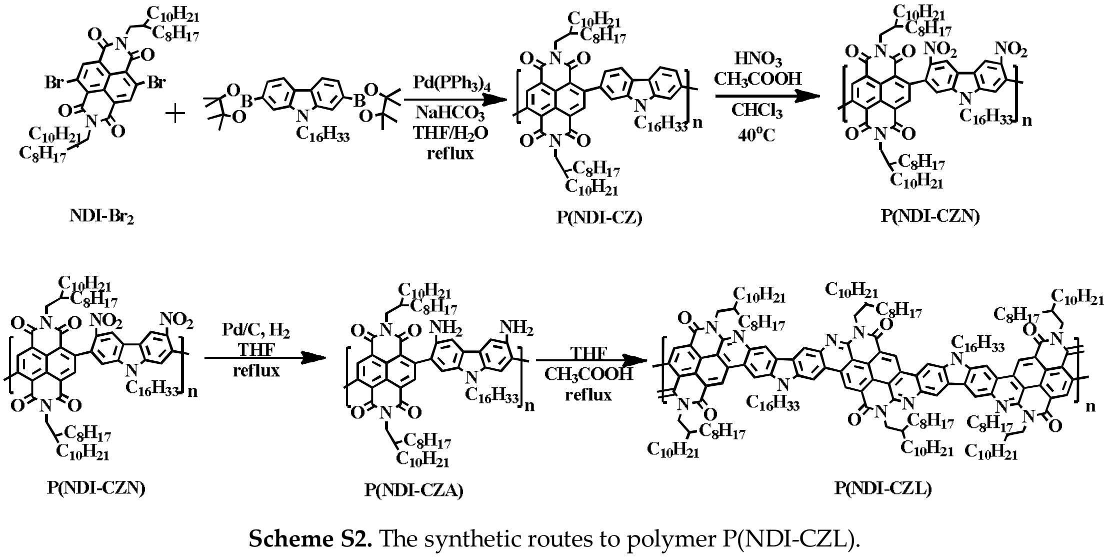

Scheme S2. The synthetic routes to polymer P(NDI-CZL).

Polymer P(NDI-CZ)

A mixture of NDI-Br_2 (1.00 g, 1.02 mmol), 2,7-bis(4,4,5,5-tetramethyl-1,3,2-dioxaborolan-2-yl)-9-hexadecyl-9H-carbazole (0.65 g, 1.02 mmol), $NaHCO_3$ (0.35 g), THF (25 mL) and H_2O (4 mL) was degassed, $Pd(PPh_3)_4$ (10 mg, 0.009 mmol) was added under a nitrogen atmosphere. The mixture was heated by microwave reactor at reflux and stirred under nitrogen for 3 h. The reaction was then cooled to room temperature, precipitated in methanol, and filtered. The solids were washed three times with methanol, water, methanol and petroleum ether, respectively. Then dried to yield the polymer product P(NDI-CZ) (1.19 g, 96%).

Polymer P(NDI-CZN)

A mixture of P(NDI-CZ) (1.10 g), concentrated nitric acid/acetic acid (2 mL, *v*/*v* = 1/3) and chloroform (10 mL) was heated at 40 °C for 24 h. The reaction was then cooled to room temperature, precipitated in methanol, and filtered. The solids were washed three times with methanol, water, methanol and petroleum ether, respectively. Then dried to yield the polymer product P(NDI-CZN) (1.13 g, 96%).

Polymer P(NDI-CZA)

A mixture of P(NDI-CZN) (1.10 g), Pd/C (70 mg) and THF (10 mL) was heated at reflux and stirred under H_2 for overnight. After cooling, Pd/C was removed by filtration, the solution was collected and removed to get the black product P(NDI-CZA) (1.03 g).

Polymer P(NDI-CZL)

A mixture of P(NDI-CZA), acetic acid (2 mL) and THF (15 mL) was heated at reflux and stirred under nitrogen for overnight. The reaction was then cooled to room temperature, precipitated in methanol, and filtered. The solids were washed three times with methanol, water, methanol and petroleum ether, respectively. Then dried to yield the polymer product P(NDI-CZL) (0.97 g, the total yield of two steps was 93%).

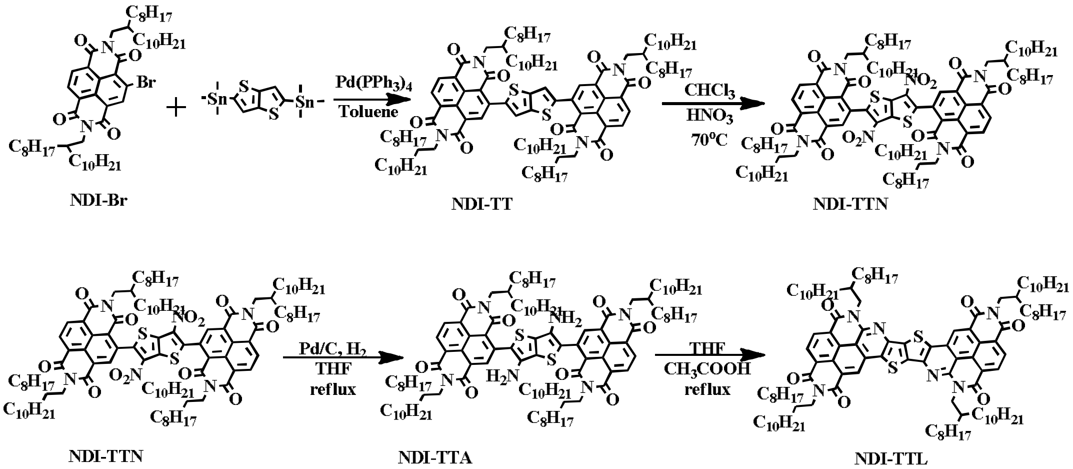

Scheme S3. The synthetic routes to compound NDI-TTL.

Compound NDI-TT

A mixture of NDI-Br (1.81 g, 2.00 mmol), 2,5-bis(trimethylstannyl) thieno [3,2-b] thiophene (0.43 g, 0.92 mmol) and toluene (4 mL) was degassed, $Pd(PPh_3)_4$ (20 mg, 0.018 mmol) was added under a nitrogen atmosphere. The mixture was heated at 100 °C and stirred under nitrogen for 3 h. After cooling, water and CH_2Cl_2 (200 mL) were added, the organic layer was separated and dried over Na_2SO_4. After removal of the solvent, the residue was chromatographically purified on silica gel eluting with petroleum ether/dichloromethane (*v*/*v* = 1/2) to afford compound NDI-TT as a dark violet solid (1.60 g, 97%). ^1H NMR (600 MHz, $CDCl_3$, ppm): δ = 8.76 (d, *J* = 7.68 Hz, 2H), 8.72 (s, 2H),

8.70 (d, *J* = 7.58 Hz,2H), 4.08 (d, *J* = 7.11 Hz, 4H), 4.04 (d, *J* = 7.29 Hz, 4H), 1.92 (m, 4H), 1.16 (m, 128H), 0.82 (t, *J* = 7.19 Hz, 24H); ^{13}C NMR (150 MHz, $CDCl_3$, ppm): δ 163.04, 162.75, 162.70, 162.25, 143.45, 141.10, 140.10, 136.14, 131.51, 130.82, 127.90, 126.34, 125.29, 123.47, 120.67, 65.72, 45.04, 44.98, 40.51, 36.57, 36.47, 31.84, 31.82, 31.62, 31.58, 31.57, 30.91, 30.01, 29.95, 29.88, 29.63, 29.56, 29.54, 29.49, 29.48, 29.26, 29.24, 29.22, 26.85, 26.61, 26.38, 26.35, 22.61, 22.59, 14.02.

Compound NDI-TTN

A mixture of NDI-TT (1.60 g, 0.90 mmol), fuming nitric acid (2 mL) and chloroform (10 mL) was heated at 70 °C for 10 h. After cooling, water and CH_2Cl_2 (200 mL) were added, the organic layer was separated and dried over Na_2SO_4. After removal of the solvent, the residue was chromatographically purified on silica gel eluting with petroleum ether/dichloromethane (*v*/*v* = 1/1) to afford compound NDI-TTN as a yellow solid (1.65 g, 98%). ^{1}H NMR (600 MHz, $CDCl_3$, ppm): δ 8.81 (d, *J* = 5.52 Hz, 4H), 8.62 (s, 2H), 4.10 (d, *J* = 6.25 Hz, 4H), 3.97 (d, *J* = 7.34 Hz, 4H), 1.94 (m, 2H), 1.82 (m, 2H), 1.18 (m, 128H), 0.79 (t, *J* = 6.38 Hz, 24H); ^{13}C NMR (150 MHz, $CDCl_3$, ppm): δ 162.75, 162.37, 162.26, 147.61, 147.54, 137.04, 137.01, 134.20, 134.07, 133.16, 132.21, 132.13, 132.08, 130.08, 129.99, 127.35, 127.31, 126.93, 126.85, 126.78, 126.72, 126.19, 126.04, 125.60, 125.47, 45.14, 45.09, 36.62, 36.60, 36.44, 36.41, 31.85, 31.83, 31.58, 31.48, 31.40. 29.97, 29.91, 29.57, 29.52, 29.47, 29.27, 29.23, 29.20, 26.36, 26.27, 22.62, 22.60, 14.03.

Compound NDI-TTA

A mixture of NDI-TTN (1.60 g, 0.85 mmol), Pd/C (70 mg) and THF (10 mL) was heated at reflux and stirred under H_2 for overnight. After cooling, Pd/C was removed by filtration, the solution was collected and removed to get the crude black product NDI-TTA (1.51 g, 98%). The compound NDI-TTA was directe to the next step without further purified.

Compound NDI-TTL

A mixture of NDI-TTA, acetic acid (2 mL) and THF (10 mL) was heated at reflux and stirred under nitrogen for overnight. After cooling, water and CH_2Cl_2 (200 mL) were added, the organic layer was separated and dried over Na_2SO_4. After removal of the solvent, the residue was chromatographically purified on silica gel eluting with petroleum ether/dichloromethane (*v*/*v* = 1/4) to afford compound NDI-TTL as a brownish red solid (1.44 g, the total yield of two steps was 95%). ^{1}H NMR (600 MHz, $CDCl_3$, ppm): δ 8.80 (s, 2H),8.41 (d, *J* = 6.75 Hz, 4H), 4.69 (d, *J* = 7.61 Hz, 4H), 4.21 (d, *J* = 5.63 Hz, 4H), 2.06 (m, 4H), 1.16 (m, 128H), 0.77 (t, *J* = 6.88 Hz, 24H); ^{13}C NMR (150 MHz, $CDCl_3$, ppm): δ 162.94, 162.46, 160.68, 147.59, 145.06, 136.08, 130.23, 129.18, 129.08, 128.60, 126.27, 126.13, 125.26, 124.90, 124.17, 123.70, 107.44, 46.16, 45.24, 36.85, 36.76, 31.87, 31.81, 31.76, 30.11, 29.67, 29.65, 29.62, 29.52, 29.32, 29.31, 29.29, 26.63, 26.52, 22.62, 22.56, 14.02, 13.99, 13.96; MS (MALDI-TOF): m/z: calcd for $C_{114}H_{170}N_6O_6S_2$: 1784.77 $[M]^+$; found: 1786.00 $[M+H]^+$.

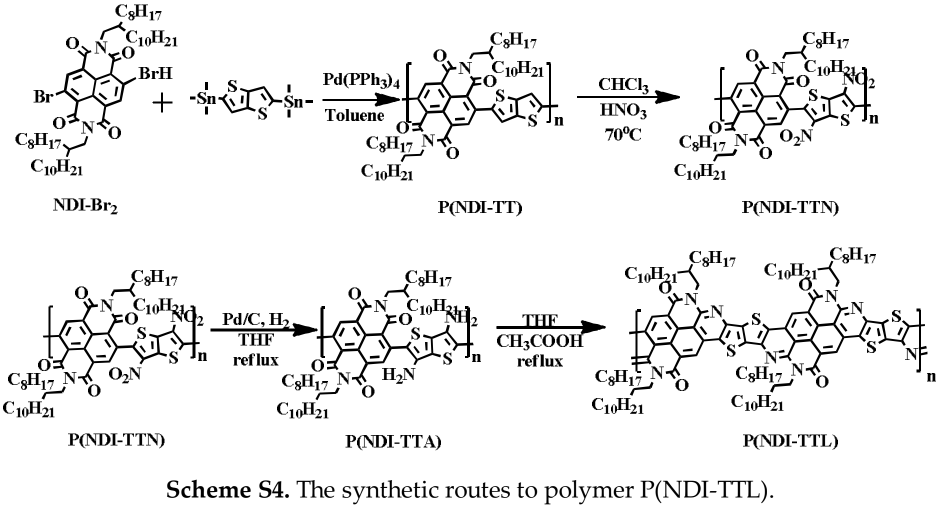

Scheme S4. The synthetic routes to polymer P(NDI-TTL).

Polymer P(NDI-TT)

A mixture of NDI-Br_2 (1.97 g, 2.00 mmol), 2,5-bis(trimethylstannyl) thieno [3,2-b] thiophene (0.93 g, 2.00 mmol) and toluene (4 mL) was degassed, $Pd(PPh_3)_4$ (20 mg, 0.018 mmol) was added under a nitrogen atmosphere. The mixture was heated at 100 °C and stirred under nitrogen for 3 h. The reaction was then cooled to room temperature, precipitated in methanol, and filtered. The solids were washed three times with methanol, water, methanol and petroleum ether, respectively. Then dried to yield the polymer product P(NDI-TT) (1.19 g, 96%).

Polymer P(NDI-TTN)

A mixture of P(NDI-TT) (1.60 g), fuming nitric acid (2 mL) and chloroform (10 mL) was heated at 70 °C for 10 h. The reaction was then cooled to room temperature, precipitated in methanol, and filtered. The solids were washed three times with methanol, water, methanol and petroleum ether, respectively. Then dried to yield the polymer product P(NDI-TTN) (1.66 g, 95%).

Polymer P(NDI-TTA)

A mixture of P(NDI-TTN) (1.60 g), Pd/C (70 mg) and THF (10 mL) was heated at reflux and stirred under H_2 for overnight. After cooling, Pd/C was removed by filtration, the solution was collected and removed to get the black product P(NDI-TTA) (1.45 g).

Polymer P(NDI-TTL)

A mixture of P(NDI-TTA), acetic acid (2 mL) and THF (15 mL) was heated at reflux and stirred under nitrogen for overnight. The reaction was then cooled to room temperature, precipitated in methanol, and filtered. The solids were washed three times with methanol, water, methanol and petroleum ether, respectively. Then dried to yield the polymer product P(NDI-TTL) (1.31 g, the total yield of two steps was 93%).

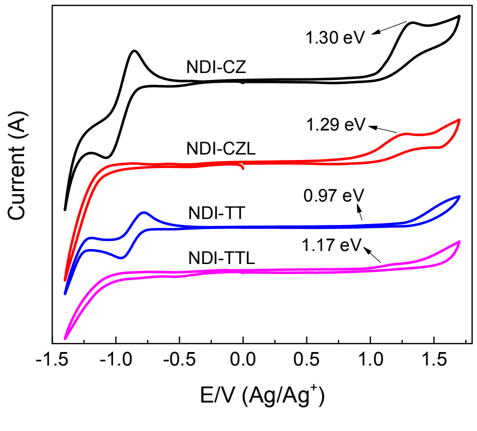

Figure S1. The cyclic voltammograms of compounds.

Table S1. the CV date of compounds.

	HOMO	LUMO	Band gap
NDI-CZ	-5.84 eV	-3.67 eV	2.17 eV
NDI-CZL	-5.72 eV	-3.84 eV	1.88 eV
NDI-TT	-5.66 eV	-3.60 eV	2.06 eV
NDI-TTL	-5.77 eV	-3.50 eV	2.27 eV

The NMR spectra and MALDI-TOF spectra

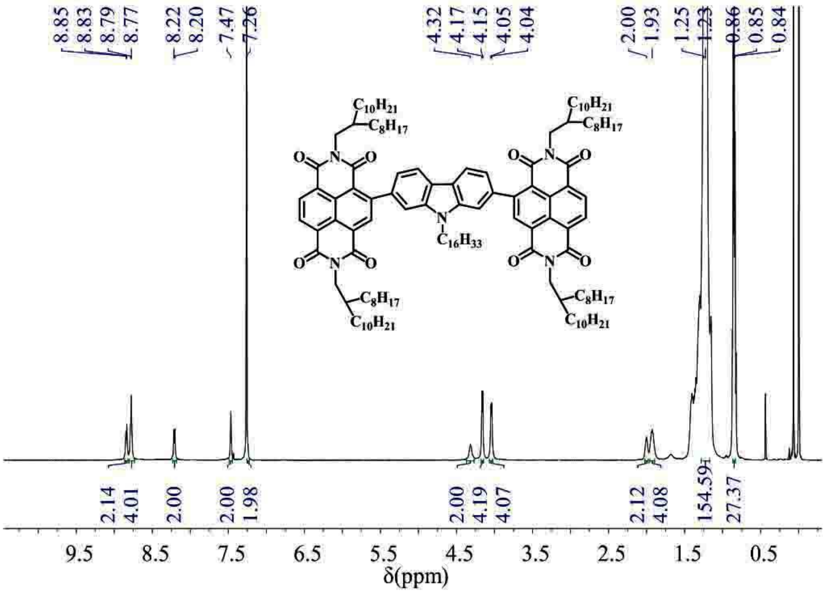

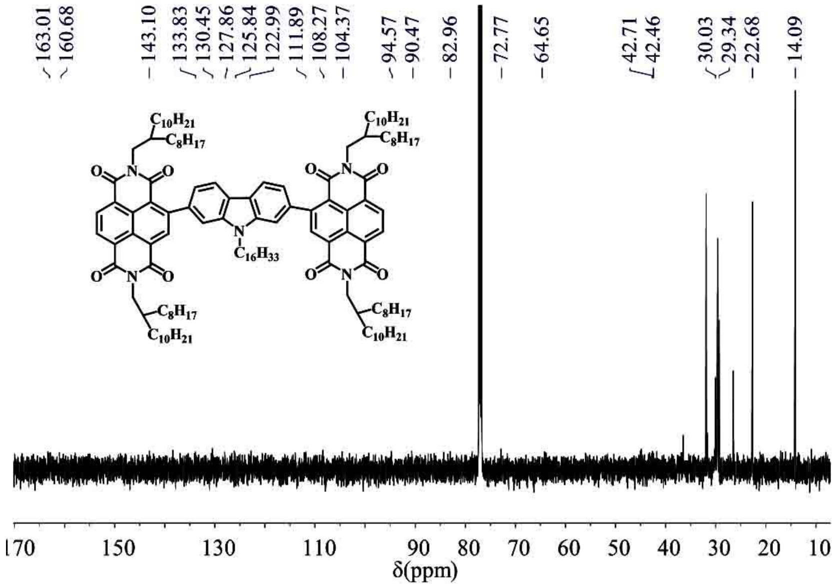

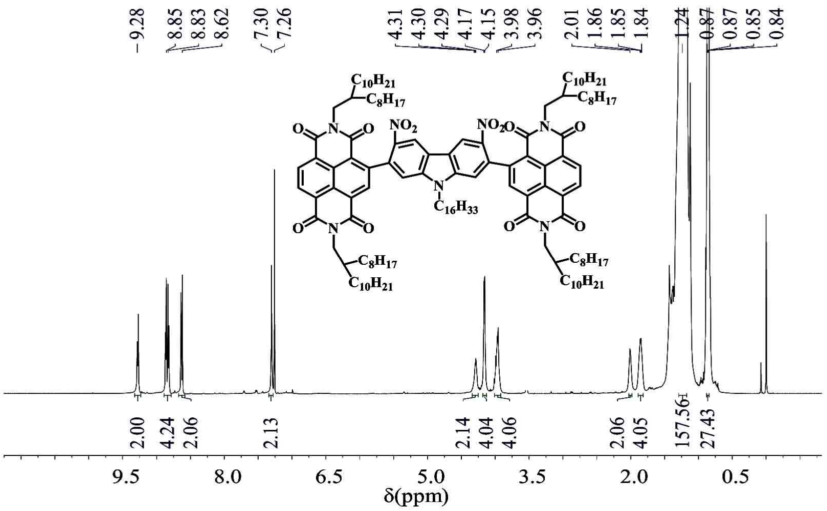

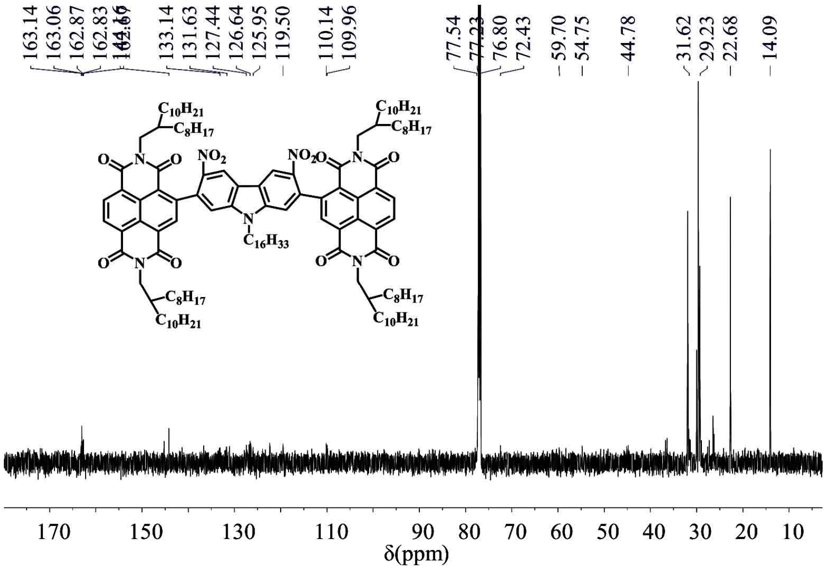
163.14
163.06
162.87
162.83
133.14
131.63
127.44
126.64
125.95
119.50
110.14
109.96
77.54
77.23
76.80
72.43
59.70
54.75
44.78
31.62
29.23
22.68
14.09
NO2
N
O
C10H21
C8H17
C16H33
170
150
130
110
90
80
70
60
50
40
30
20
10
δ(ppm)

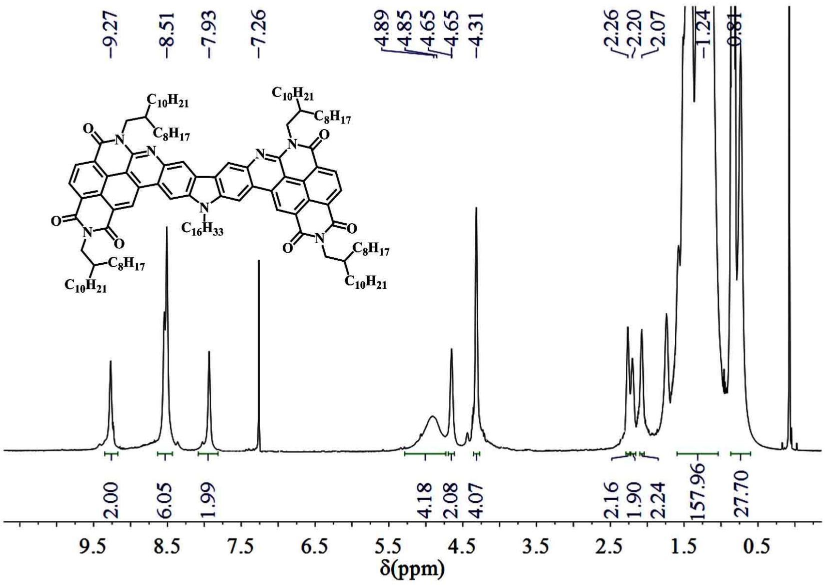
9.27
8.51
7.93
7.26
4.89
4.85
4.65
4.65
4.31
2.26
2.20
2.07
1.24
0.81
2.00
6.05
1.99
4.18
2.08
4.07
2.16
1.90
2.24
157.96
27.70
C10H21
C8H17
C16H33
9.5
8.5
7.5
6.5
5.5
4.5
3.5
2.5
1.5
0.5
δ(ppm)

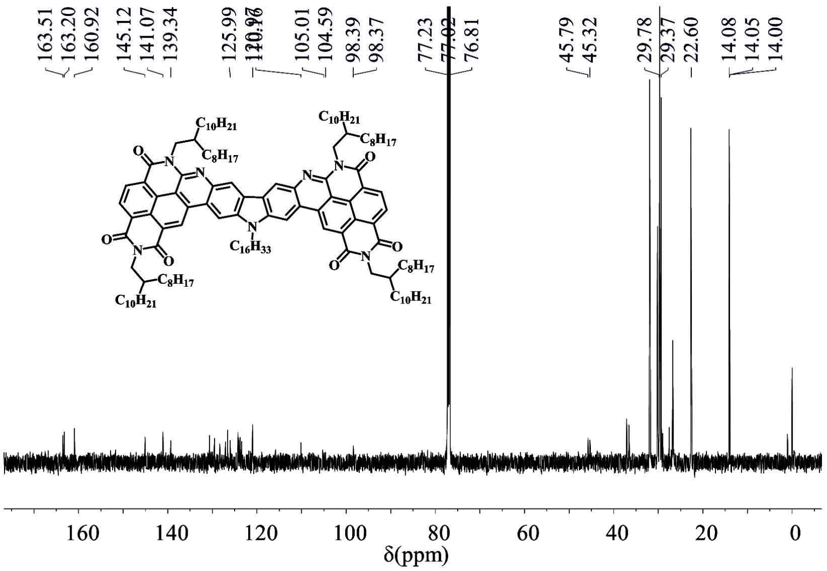
163.51
163.20
160.92
145.12
141.07
139.34
125.99
105.01
104.59
98.39
98.37
77.23
76.81
45.79
45.32
29.78
29.37
22.60
14.08
14.05
14.00
C10H21
C8H17
C16H33
160
140
120
100
80
60
40
20
0
δ(ppm)

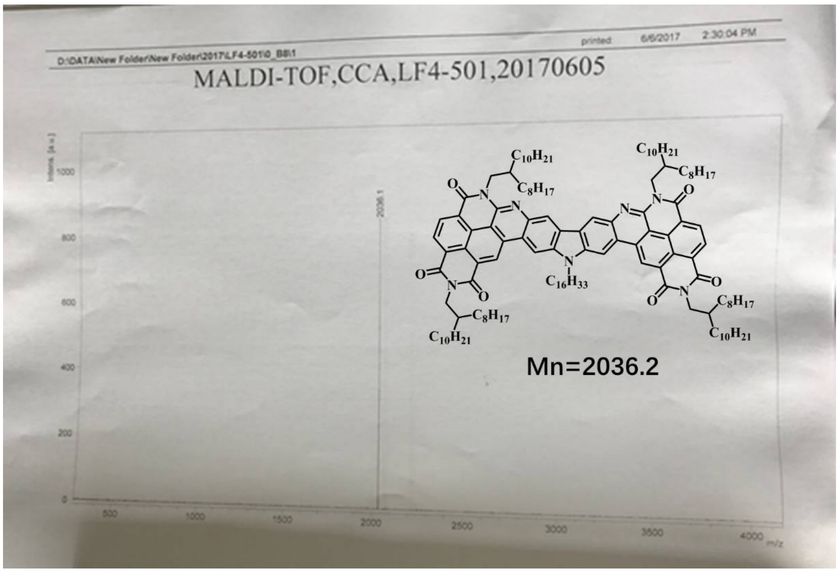
D:\DATA\New Folder\New Folder\2017\LF4-501\0_B8\1
printed 6/6/2017 2:30:04 PM
MALDI-TOF,CCA,LF4-501,20170605
2036.1
Mn=2036.2
500 1000 1500 2000 2500 3000 3500 4000 m/z

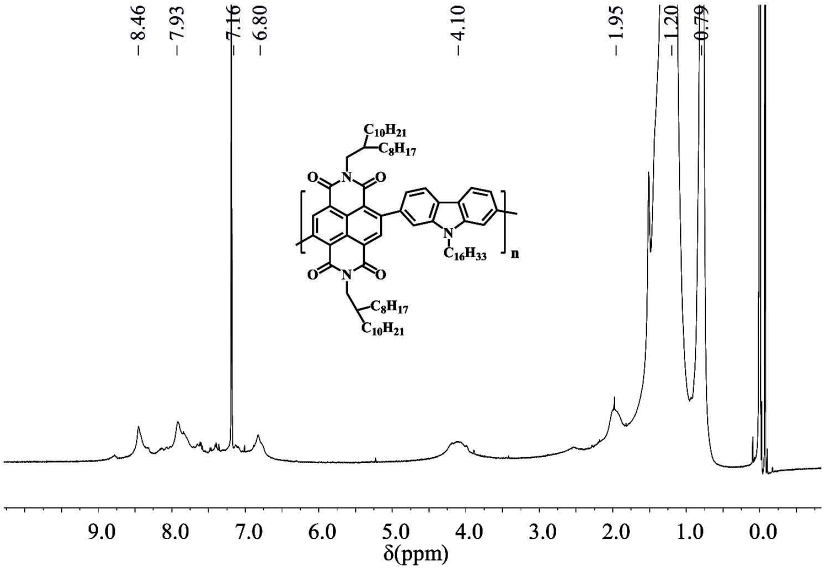
8.46 7.93 7.16 6.80 4.10 1.95 1.20 0.79
9.0 8.0 7.0 6.0 5.0 4.0 3.0 2.0 1.0 0.0
δ(ppm)

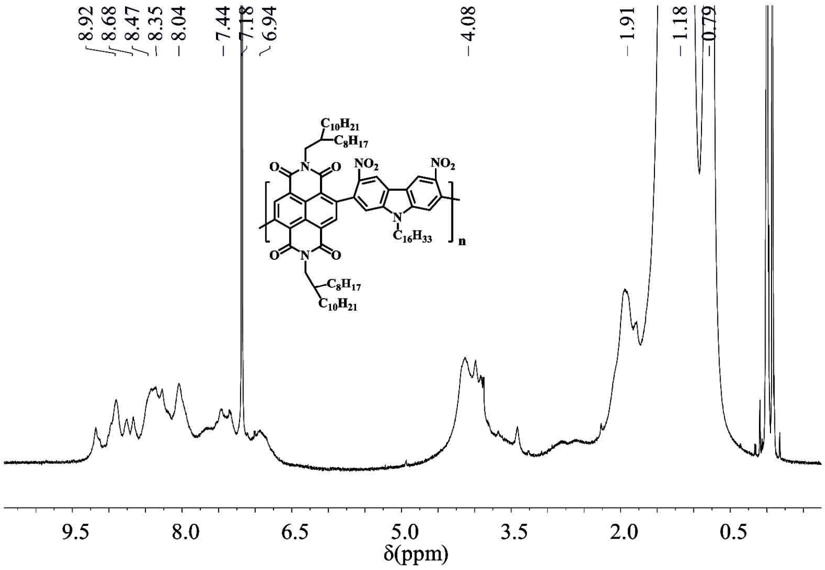
8.92 8.68 8.47 8.35 8.04 7.44 7.18 6.94 4.08 1.91 1.18 0.79
9.5 8.0 6.5 5.0 3.5 2.0 0.5
δ(ppm)

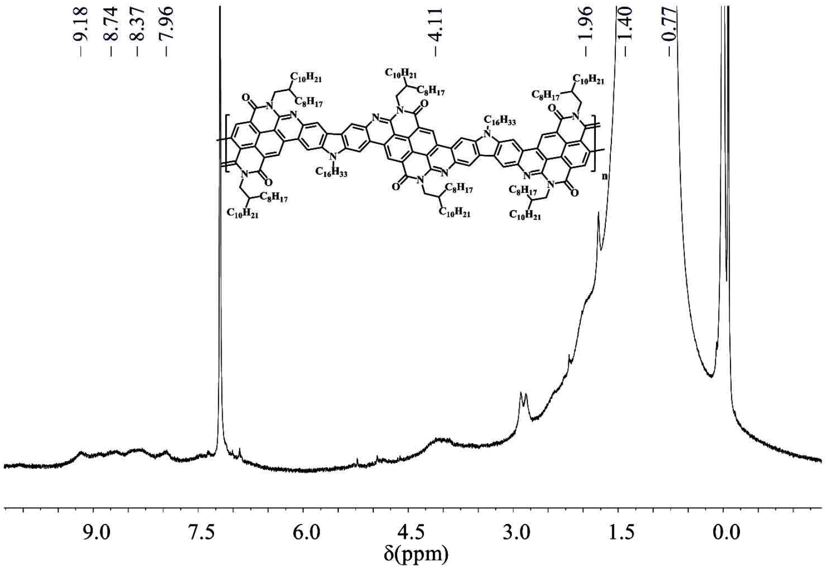
9.18
8.74
8.37
7.96
4.11
1.96
1.40
0.77
9.0
7.5
6.0
4.5
3.0
1.5
0.0
δ(ppm)

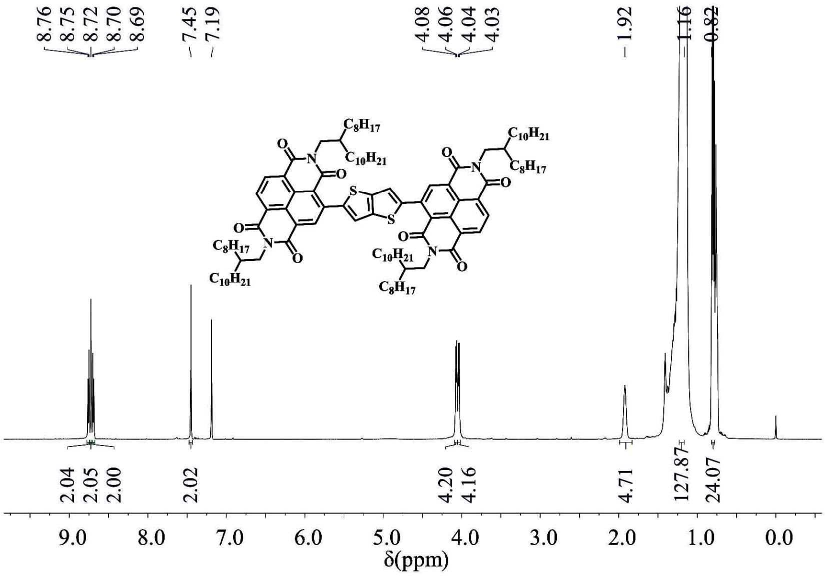
8.76
8.75
8.72
8.70
8.69
7.45
7.19
4.08
4.06
4.04
4.03
1.92
1.16
0.82
2.04
2.05
2.00
2.02
4.20
4.16
4.71
127.87
24.07
9.0
8.0
7.0
6.0
5.0
4.0
3.0
2.0
1.0
0.0
δ(ppm)

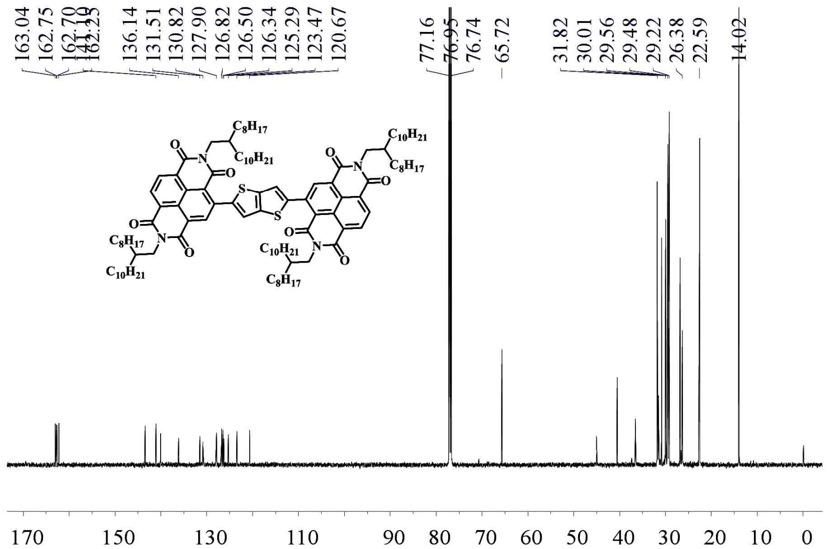
163.04
162.75
162.70
136.14
131.51
130.82
127.90
126.82
126.50
126.34
125.29
123.47
120.67
77.16
76.74
65.72
31.82
30.01
29.56
29.48
29.22
26.38
22.59
14.02
170
150
130
110
90
80
70
60
50
40
30
20
10
0
?(ppm)

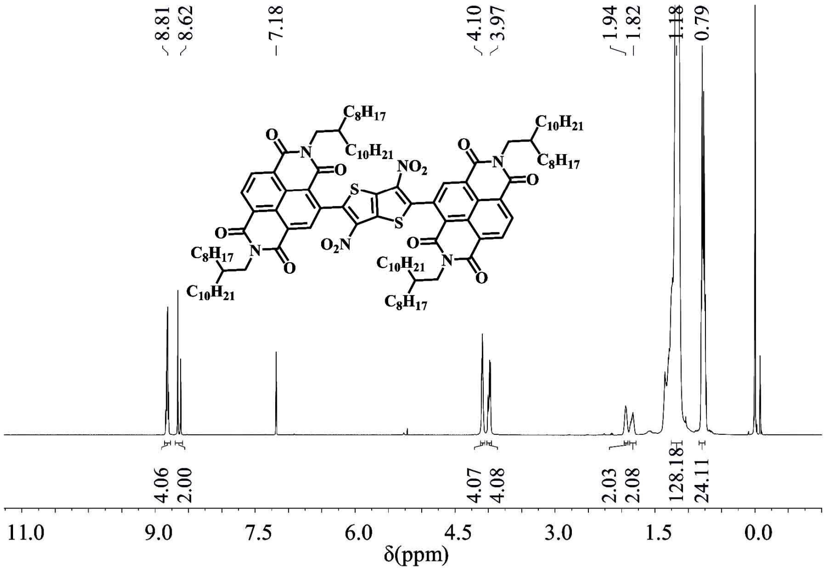
8.81
8.62
7.18
4.10
3.97
1.94
1.82
1.18
0.79
4.06
2.00
4.07
4.08
2.03
2.08
128.18
24.11
11.0
9.0
7.5
6.0
4.5
3.0
1.5
0.0
δ(ppm)

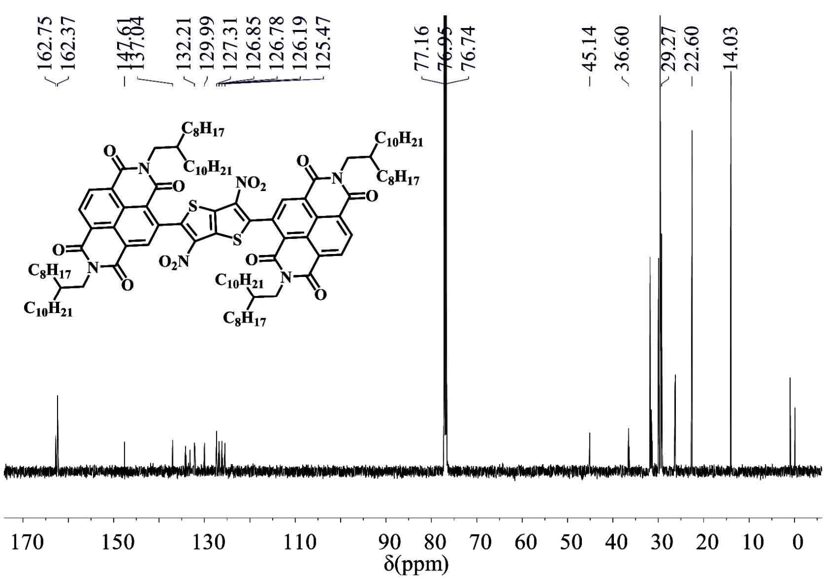
162.75
162.37
132.21
129.99
127.31
126.85
126.78
126.19
125.47
77.16
76.74
45.14
36.60
29.27
22.60
14.03
170
150
130
110
90
80
70
60
50
40
30
20
10
0
δ(ppm)

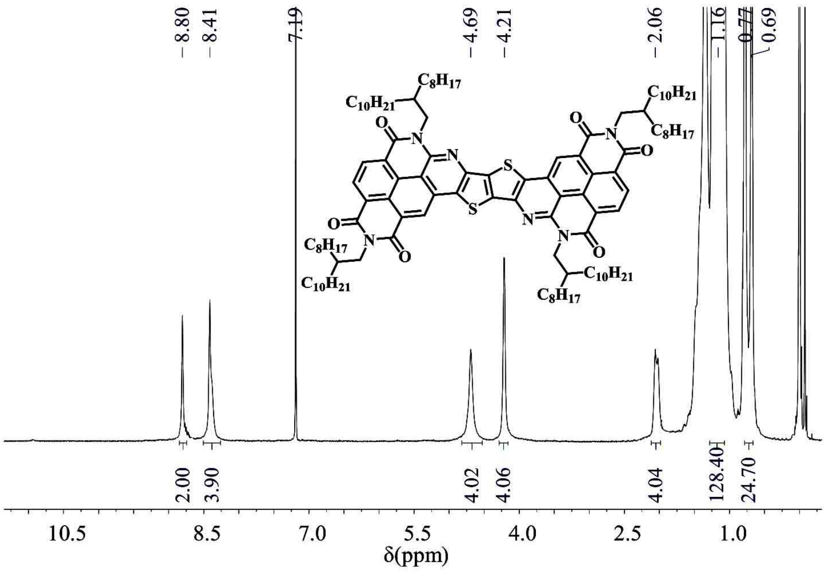
8.80
8.41
4.69
4.21
2.06
1.16
0.69
2.00
3.90
4.02
4.06
4.04
128.40
24.70
10.5
8.5
7.0
5.5
4.0
2.5
1.0
δ(ppm)

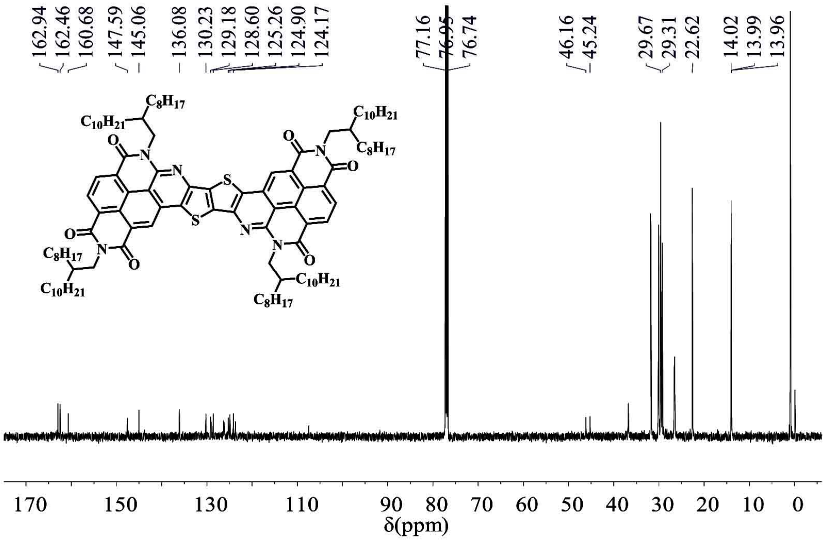
162.94
162.46
160.68
147.59
145.06
136.08
130.23
129.18
128.60
125.26
124.90
124.17
77.16
76.95
76.74
46.16
45.24
29.67
29.31
22.62
14.02
13.99
13.96
C_8H_{17}
$C_{10}H_{21}$
170 150 130 110 90 80 70 60 50 40 30 20 10 0
δ(ppm)

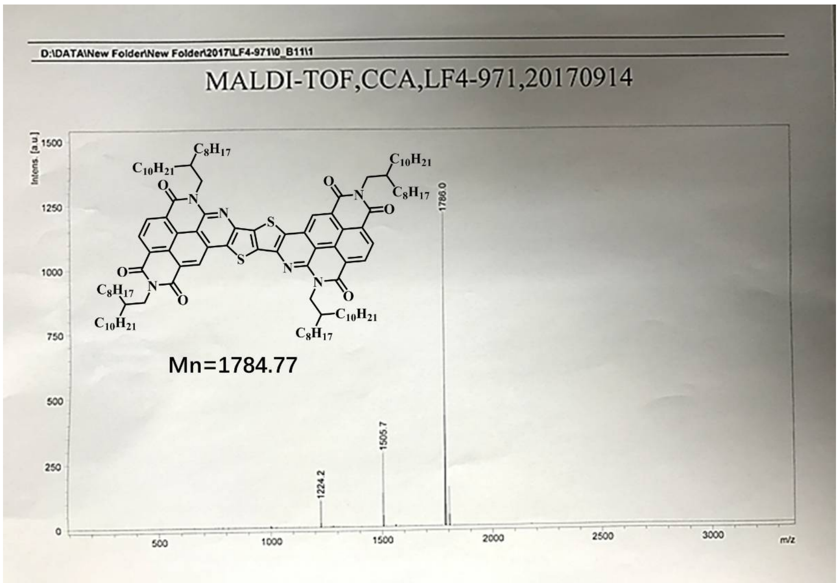
MALDI-TOF,CCA,LF4-971,20170914
C_8H_{17}
$C_{10}H_{21}$
Mn=1784.77
1786.0
1505.7
1224.2
m/z

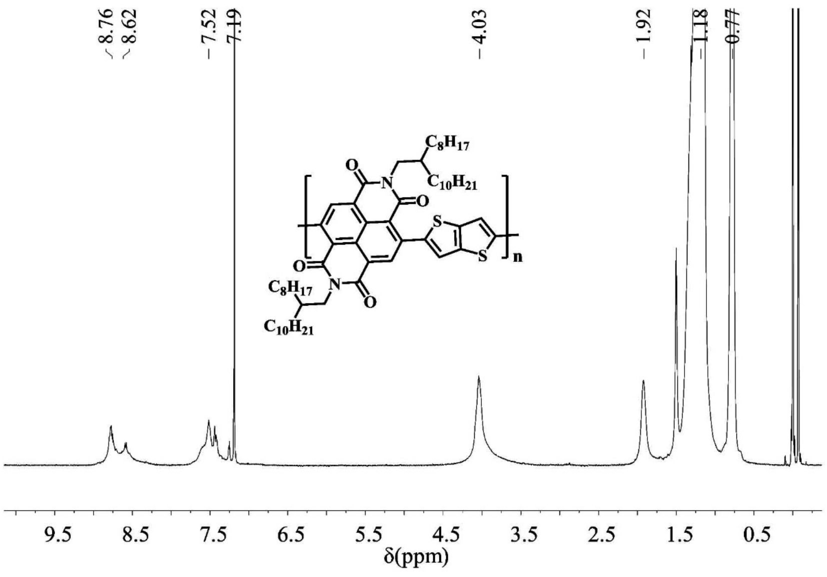
8.76
8.62
7.52
7.19
4.03
1.92
1.18
0.77
C_8H_{17}
$C_{10}H_{21}$
n
9.5 8.5 7.5 6.5 5.5 4.5 3.5 2.5 1.5 0.5
δ(ppm)

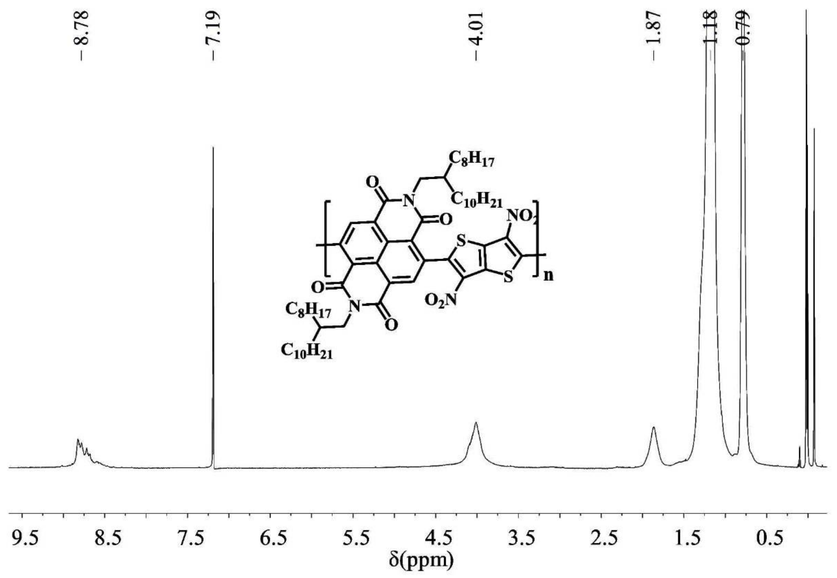

8.78
7.19
4.01
1.87
1.18
0.79
C8H17
C10H21
NO2
O2N
n
9.5
8.5
7.5
6.5
5.5
4.5
3.5
2.5
1.5
0.5
δ(ppm)

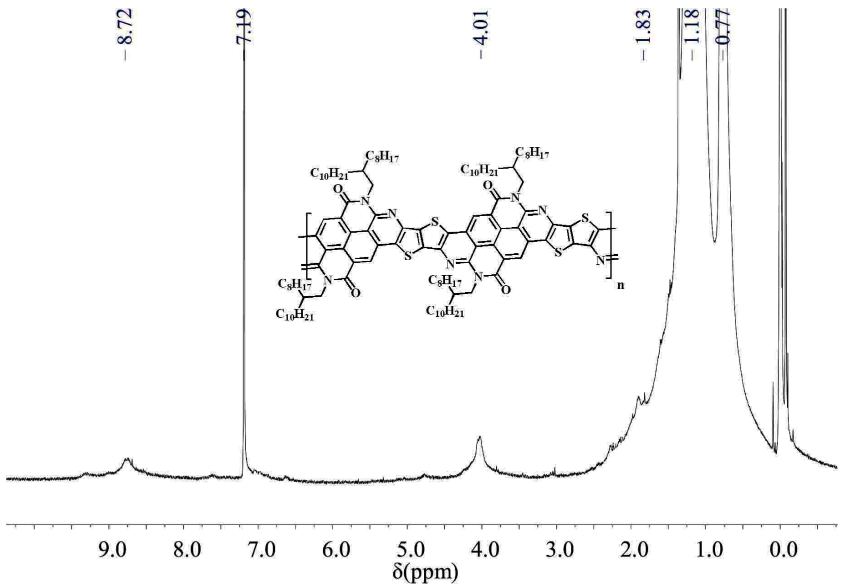

8.72
7.19
4.01
1.83
1.18
0.77
C8H17
C10H21
n
9.0
8.0
7.0
6.0
5.0
4.0
3.0
2.0
1.0
0.0
δ(ppm)
